# Pharmacokinetic, pharmacodynamic, and pharmacogenetic assays to monitor clopidogrel therapy

**DOI:** 10.1002/prp2.686

**Published:** 2020-11-17

**Authors:** Bhawani Yasassri Alvitigala, Lallindra Viranjan Gooneratne, Godwin Roger Constantine, Rajapaksha Arachchige Namal Kumarasiri Wijesinghe, Liyanage Dona Ashanthi Menuka Arawwawala

**Affiliations:** ^1^ Department of Medical Laboratory Science Faculty of Health Sciences The Open University of Sri Lanka Nugegoda Sri Lanka; ^2^ Department of Pathology Faculty of Medicine University of Colombo Colombo Sri Lanka; ^3^ Department of Clinical Medicine Faculty of Medicine University of Colombo Colombo Sri Lanka; ^4^ Department of Clinical Sciences Faculty of Medicine General Sir John Kotelawala Defense University Ratmalana Sri Lanka; ^5^ Research & Development Complex Industrial Technology Institute Malabe Sri Lanka

**Keywords:** clopidogrel, HPLC, platelet function assays, pharmacogenetic assays

## Abstract

Clopidogrel is the most common and widely used antiplatelet agent for patients with coronary artery disease following confirmation by electrocardiographic studies. The nonresponsiveness of patients to clopidogrel and the possibility of testing for clopidogrel resistance by platelet function assays (PFA) are contentious issues. Light transmission aggregometry (LTA) is considered as the gold standard test among all PFA. In this review, the most commonly used PFA used for monitoring the effect of clopidogrel, LTA, vasodilator‐stimulated phosphoprotein assay phosphorylation, rotational thromboelastometry (ROTEM) delta and ROTEM platelet, thromboelastography, PFA‐100, VerifyNow P2Y12 assay, Multiplate analyzer, Plateletworks assay and pharmacogenetic studies, are comparatively discussed including their principles of action, advantages, and disadvantages. VerifyNow P2Y12 assay can be accepted as the ideal point of care test out of the discussed assays. However, modified assays are required which could overcome the limitations associated with currently available assays.

## Graphical abstract

This review article discuss the principles of pharmacokinetic, pharmacodynamic and pharmacogenetic assays used for monitoring clopidogrel therapy and comparatively discuss their advantages and disadvantages.

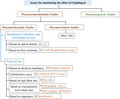

**Abbreviations**
ADP: adenosine diphosphate
APTT: activated partial thromboplastin time
AU: aggregation units
CAD: coronary artery disease
CADP: collagen‐ADP
cAMP: cyclic adenosine monophosphate
CFT: clot formation time
CLPM: clopidogrel metabolite
CTM: converted to thiol metabolite of clopidogrel
EDTA‐K: ethylenediaminetetraacetic potassium salt
HPLC: high‐performance liquid chromatography
HTPR: high on‐treatment platelet reactivity
INR: international normalized ratio
LTA: light transmission aggregometry
MCF: maximum clot firmness
MFI: mean fluorescence intensity
ML: maximum lysis
MPV: mean platelet volume
MS: mass spectrometry
NSAID: nonsteroidal anti‐inflammatory drug
PCI: percutaneous coronary intervention
PFA: platelet function assay
PGE1: prostaglandin E1
POCT: point of care test
PRI: platelet reactivity index
PRP: platelet‐rich plasma
PRU: platelet reactivity units
PT: prothrombin time
ROTEM: rotational thromboelastometry
STEMI: ST‐elevated myocardial infarction
TEG: thromboelastography
UHPLC: ultra‐high‐performance liquid chromatography
VASP: vasodilator‐stimulated phosphoprotein
vWF: von Willebrand factor

## 1 INTRODUCTION

Clopidogrel is a second‐generation thienopyridine drug which acts as an inhibitor of platelet aggregation and hence used as an effective medication for coronary artery disease (CAD) and percutaneous coronary intervention (PCI).^1^, ^2^ However, clopidogrel resistance among patients has become a significant concern. Hence, it has become important to decide whether the patient is clopidogrel resistant or not.^3^ If resistant, then the optimum dose or changing/adding a different antiplatelet drug needs to be considered. In order to resolve this problem, platelet function tests (PFTs) play an important role in providing information for physicians regarding management of patients on clopidogrel therapy. This article reviews the role of platelet function assays (PFA) in monitoring clopidogrel therapy, together with the principles, applications, and limitations of the different tests discussed comparatively. Further, the future prospects of PFTs are also discussed.

## 2 ACTION OF CLOPIDOGREL

Antiplatelet therapy is an essential pharmacological therapy given to patients with atherothrombotic disease to inhibit the platelet aggregation by blocking the platelet receptors involved in adhesion. Genetic variations between patients and delayed onset of action of clopidogrel result in the increase in resistance to clopidogrel therapy. Major reason for clopidogrel resistance was identified as the cytochrome P450 CYP2C19 polymorphism.^4^, ^5^

**FIGURE 1:**
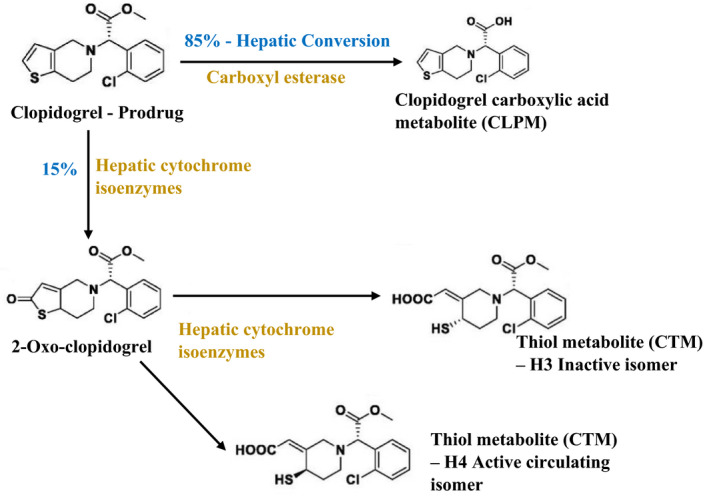
Metabolic activation of clopidogrel prodrug in patients. CLPM, clopidogrel metabolite; CTM, converted to thiol metabolite of clopidogrel

When clopidogrel is ingested, 85% of prodrug is absorbed by liver and converted to its carboxylic acid derivative by carboxylic esterase. This is known as CLPM (clopidogrel metabolite), which is a major inactive metabolic product circulating in the blood and helps to determine the pharmacokinetics of the prodrug. Fifteen percent will be converted to thiol metabolite of clopidogrel (CTM) by the hepatic cytochrome isoenzymes (CYP P450 1A2, CYP2B6, CYP2C9, CYP2C19, and CYP3A4). CTM involves in specific and irreversible blocking of P2Y12 receptors, inhibiting the adenosine diphosphate (ADP)‐induced platelet aggregation. CTM consists of four isomers; H1‐H4, where H3 (inactive form) and H4 (active circulating form) are mainly considered in monitoring the action of clopidogrel (Figure 1).^6^, ^7^, ^8^, ^9^, ^10^, ^11^

Thiol will irreversibly bind to P2Y12‐ADP receptor on platelets via a permanent disulfide bond with two cysteine residues (cys 17 and cys 270) on the receptor, thus inhibiting the ADP binding permanently and thereby activating adenylyl cyclase enzyme to increase cyclic adenosine monophosphate (cAMP). Thus, protein kinase level increases, stimulating phosphorylation of vasodilator‐stimulated phosphoprotein (VASP). Hence, inhibiting the activation of receptor complex gp IIb/III. Consequently, no thrombosis will occur. Furthermore, clopidogrel reduces secretions from dense granules in platelets and reduces arachidonic acid, collagen, and thrombin‐induced platelet activation. Clopidogrel also decreases the enzymatic activation of coagulation pathway, decreasing thrombin formation. Clopidogrel action solely depends on dose and time. Usually, 400‐600 mg of dose is given and will take 2‐5 hours for the platelet inhibition with 400 mg dose. Seventy‐five milligram of daily dose will take about 7 days for maximum platelet inhibition and management of atrial fibrillation. Half‐life of clopidogrel active metabolite is approximately 6 hours.^12^, ^13^, ^14^, ^15^

## 3 CLOPIDOGREL RESISTANCE

Although clopidogrel is widely used, it shows increased resistance/response variability among thrombosis patients as it is highly susceptible to drug interactions and CYP gene single nucleotide polymorphisms, thus reducing the in vitro enzyme activity inhibiting/reducing the conversion of clopidogrel into its active thiol metabolites. Therefore, it is difficult to decide the relevant dose without performing a PFT.^16^, ^17^ Degree of clopidogrel resistance may vary based on the laboratory method used.^14^ Prevalence of resistance with clopidogrel and aspirin has been studied by using different laboratory techniques. Expected prevalence of aspirin resistance by those studies was about 5.5% to 60%, while clopidogrel resistance was 16.8% to 21%. Hence, dual antiplatelet therapy or triple therapy along with another potent agent was required.^18^ Clopidogrel resistance is mainly associated with CYP2C19*2 genotype loss‐of‐function allele. Genetic variations regarding conversion to active metabolite by CYP P450 enzymes also play a major role in resistance.^17^, ^19^, ^20^ Conferring to the studies, clopidogrel resistance in population was about 4%‐30%, and the variation was due to the use of different platelet function studies.^21^, ^22^, ^23^ A study on clopidogrel resistance in India using optical aggregometry has shown that, in the population, 2.54% were resistant, 12.7% were semi‐responders, and 84.7% were responders to clopidogrel.^24^ There are several underlying causes for clopidogrel resistance. Age, gender, obesity, long‐term medications, and stress, such as individual factors, may contribute to clopidogrel resistance. Mainly, genetic factors such as mutations in P2Y12 gene, P450 CYP3A gene, and COX1 gene and polymorphism in platelet glycoprotein receptors affect the resistance. Further, overproduction of platelets by bone marrow, transfusion of platelets, failure in compliance, insufficient dose, and absorption by hepatic cells and certain drug interactions, such as nonsteroidal anti‐inflammatory drugs (NSAIDs), will also result in clopidogrel resistance.^25^, ^26^, ^27^

## 4 PLATELET FUNCTION TESTS TO MONITOR CLOPIDOGREL THERAPY

Platelet function tests play a major role in determining the effect of antiplatelet drugs, such as clopidogrel, on inhibiting platelet aggregation. Although light transmission aggregometry (LTA) is considered as gold standard, it has many limitations. PFTs should possess several features for them to be ideal in use. They should be cost‐effective, less labor expertise, high sensitivity and specificity, produce quick results, and define the clinical situation more precisely. Further, they should have standardized test procedure with proper quality control, reference ranges to quantify the antiplatelet effect, and measure nonresponsiveness to antiplatelet drug, specific for the platelet receptor to measure the drug and its active metabolites. Importantly, they should have the capacity to monitor platelet activation‐induced physiologically relevant agonists and to detect high‐risk and low‐risk patients for antiplatelet drug resistance.^28^

However, when most PFTs are considered they are less sensitive and cannot be performed in relation to normal physiological state. The reason is that, PFTs are performed on anticoagulated whole blood with relatively high concentrations of agonists including platelet aggregation. Further, the platelets will form more stabilized thrombus at high shear rate, which is difficult to be achieved by PFTs. Normal pathological shear rate is >10 000 s^−1^.^29^ At low shear rate of PFT, the effect of clopidogrel inhibitor is overrated, as the inhibitor fails to ban the platelet deposition on reactive surfaces due to shear gradient.^30^ In addition, PFTs have different cut‐off values to define the clopidogrel nonresponsiveness, hence it has become problematic to build up better correlation of results between different PFTs with different principles.^31^

Most basic laboratory test is full blood count, where the mean platelet volume (MPV) parameter implies the platelet activation and clopidogrel resistance.^32^ ADP is the commonly used agonist for monitoring clopidogrel action by PFTs. There are two ADP receptors on the surface of platelets which involve in platelet aggregation. P2Y1 receptor stimulates ADP‐induced platelet aggregation via phospholipase C and phosphatidylinositol pathways. Second receptor is P2Y12, which stimulates platelet activation by inhibiting adenylyl cyclase signaling pathway. P2Y12 receptor essentially plays a role in producing more stabilized ADP‐induced platelet aggregation. Hence, the clopidogrel therapy mainly targets on blocking this P2Y12 receptors.^33^

Platelet function tests are affected by many pre‐analytical variables, such as operator errors and phlebotomy errors, which may cause damage to the vessel walls inducing platelet coagulation. Other factors which affect the PFT results are age, gender, and clinical conditions (acquired platelet disorders, vWF, Hermansky‐Pudlak syndrome, and Chediak‐Higashi syndrome), certain foods—garlic, turmeric, and cumin seeds—and drugs such as aspirin, aspirin‐containing drugs, NSAIDs, tricyclic antidepressants, some antibiotics, and antihistamines, and other long‐term medications.^34^

Prior to the PFT, the anticoagulated whole blood sample of the patient should be tested for full blood count/platelet count, to exclude pseudothrombocytopenia due to cold platelet agglutinin, and MPV, to identify high MPV which indicates platelet turnover and low values indicate bone marrow failure. Blood film and morphology will be used to confirm the conditions, such as primary platelet disorders (Gray platelet syndrome), presence of platelet clumps (pseudothrombocytopenia), and thrombocytopenia.^35^, ^36^, ^37^

Plasma sample should be tested for prothrombin time (PT), international normalized ratio (INR), activated partial thromboplastin time (APTT), and fibrinogen levels to check the fibrinogen levels as they are important for platelet aggregation.^36^, ^37^ Collected samples should be performed within 30‐90 minutes, which is known as the turnaround time, and be controlled by minimizing the sample transportation time, centrifugation time, and increasing the calibration ranges. It is vital to perform PFT immediately in the case of serious bleeding, >6 units of red cells in 12 hours.^35^

### 4.1 Anticoagulants used for PFTs

Except for high‐performance liquid chromatography (HPLC), which requires whole blood collected in ethylenediaminetetraacetic potassium salt (EDTA‐K), other PFA could be performed on blood collected in 3.2% trisodium citrate anticoagulant at room temperature and physiological pH. Agonists play an important role by increasing the intracellular/cytosolic Ca^2+^, thereby inducing the platelet activation. Sodium citrate has the ability to chelate the extracellular calcium (Ca^2+^). Hence, citrate will have a slight chance to chelate the intracellular Ca^2+^ as well as causing decreased platelet aggregation when the samples are taken for analysis 4 hours after collection. Further, when citrated blood is used, blood sample becomes highly nonphysiological and has reduced the accuracy of results obtained via thromboelastogram (TEG).^38^ When citrated blood is used, the degree of platelet aggregation will not be changed up to 2 hours; however, the aggregation gradually decreases thereafter. Even small rise in quantity of sodium citrate will show significant inhibition of platelet aggregation along with ADP, and collagen as sodium citrate acts as the major agent associated with ADP‐induced aggregation. At low plasma Ca^2+^ (0.1 mmol/L), citrated blood fails to generate thrombin, thus reduces the growth and stability of thrombus. Generally, 0.25 ± 0.05 mmol/L free calcium is needed for thrombin generation. Thereby, citrated blood seem to affect the reproducibility and sensitivity of the assay to monitor the ADP‐induced platelet aggregation in patients under clopidogrel therapy.^39^, ^40^

**FIGURE 2:**
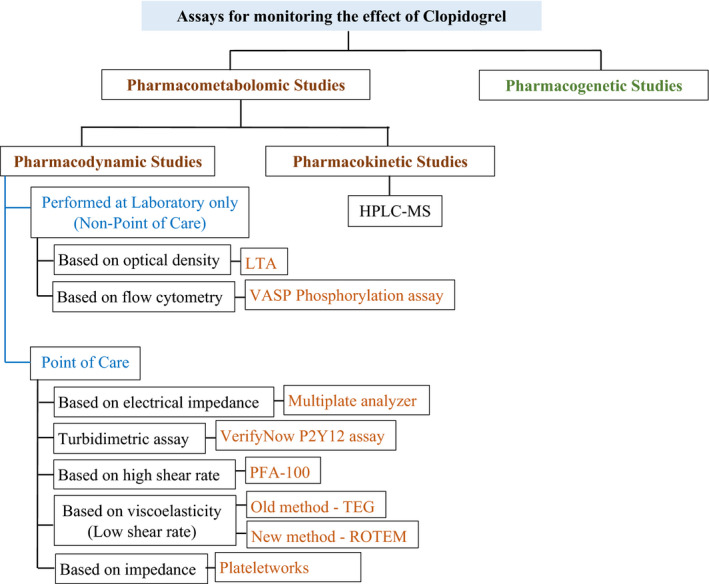
PFA available to monitor the effect of clopidogrel in patients with CAD. HPLC‐MS, high performance liquid chromatography‐mass spectrometry; LTA, light transmission aggregometry; PFA, platelet function assay; ROTEM, rotational thromboelastometry; TEG, thromboelastography; VASP, vasodilator‐stimulated phosphoprotein

In order to overcome the faults, hirudin anticoagulant was introduced, which is extracted from leeches. Hirudin can directly inhibit the thrombin without changing the Ca^2+^ levels in the sample. Since thrombin is inhibited, coagulation of the blood sample was also inhibited as a result of blocking the conversion of fibrinogen to fibrin. Further, hirudin can maintain the ADP levels in the samples for more than 4 hours. In addition, the poor platelet impedance observed in citrated blood due to the effect from collagen and ADP was resolved when using hirudin anticoagulant. Moreover, Multiplate assay requires tight platelet aggregation for a remarkable change in impedance, which could not be strongly detected by citrated samples. Hence, hirudin is highly recommended for use while lepirudin, phenylalanyl‐L‐prolyl‐L‐arginine chloromethyl ketone also can be used.^41^, ^42^, ^43^ The assays (Figure 2) that are available to monitor the effect of clopidogrel on platelet activity are discussed comparatively under this review.

### 4.2 Assays performed in the laboratory (non‐point of care)

#### Light transmission aggregometry

Light transmission aggregometry is considered as the gold standard test performed on either whole blood or platelet‐rich plasma (PRP) and is used worldwide. The platelet function is measured by measuring the change in optical density when light passes through the PRP after the addition of the agonists, commonly used as ADP, arachidonic acid, collagen, epinephrine, and thrombin. ADP is regarded as the most common agonist to determine the effect of clopidogrel on inhibiting platelet aggregation. Low‐dose ADP (1, 2.5, or 5 µm) will initially bind to P2Y1 receptors on the platelet, inducing intracellular calcium efflux. Hence, results in altering the shape of platelets causing primary wave of platelet aggregation. Secondary wave is formed due to the release of ADP from platelet storage granules. Low‐dose ADP induces only the reversible primary wave. ADP has the capacity to bind for P2Y12 receptors. When high concentration of ADP agonist (10 and 20 µm) is added, it will bind to P2Y12 receptors, initiating the platelet aggregation, without changing the shape. P2Y12 receptor is the major receptor for ADP and is responsible for complete platelet aggregation. However, if the patient is a better clopidogrel responder, clopidogrel will block the binding of ADP to P2Y12 receptors, thus preventing the second wave of platelet aggregation. Such that, the percentage of light transmission in PRP will provide the percentage of maximum platelet aggregation (% MPA) and percentage inhibition of platelet aggregation (% IPA). When light passes through PRP, initially considered platelet aggregation is 0% (no aggregation), then the light transmission through the PRP is 0% (hence, 100% inhibition by clopidogrel). If aggregation initiates upon the addition of agonist, light transmission too increases. At platelet poor plasma (PPP), it is considered that 100% light transmission for 100% platelet aggregation (hence, 0% inhibition by clopidogrel). It could be interpreted that the decreased light transmission indicates the better clopidogrel responders. In order to overcome the limitations of above conventional method, a new method was developed where PRP was incubated with prostaglandin E1 (PGE1). Once pretreated with PGE1, prior to addition of ADP, the effect of P2Y1 receptors will be completely inhibited. Common ADP agonist doses are 5, 10, or 20 µmol/L. LTA is affected by pre‐analytical variables, such as anti‐inflammatory drugs, food stuff like garlic, turmeric, and caffeine, and high fat diet, and should adjust the count within 200‐400 × 109/L. If high, adjust with PPP. Counts less than the range will reduce the aggregation responses.^22^, ^44^, ^45^, ^46^, ^47^, ^48^ Cut‐off value for suboptimal clopidogrel response in terms of percentage aggregation is ≥ 70% for 10µM ADP and ≥ 50% for 5µM ADP^49^

#### Vasodilator‐stimulated phosphoprotein assay

Vasodilator‐stimulated phosphoprotein is an actin regulatory protein and a substrate for both cAMP and cGMP (cyclic guanosine monophosphate)‐dependent protein kinases and involves in the filopodia formation and adhesion of platelets. When P2Y12 receptors are blocked by clopidogrel and PGE1 is stimulated, activated adenylyl cyclase will initiate the VASP phosphorylation. Hence, VASP phosphorylation will indicate the P2Y12 inhibition by clopidogrel.^50^, ^51^ However, still the direct relationship between the VASP phosphorylation and ADP‐induced in vivo platelet aggregation has not yet described.^52^

Citrated anticoagulant blood samples are used along with the commercially available kit included with ADP and PGE1. Assay is based on the flow cytometric technique. Upon incubation of platelets with PGE1 and ADP, platelets will be reacted with CD61 phycoerythrin‐labeled platelet‐specific antibody and FITC‐labeled phosphorylated VASP‐specific mouse monoclonal antibody. The used antibody is specific for the phosphorylated form of VASP. Likely, pretreated samples will be analyzed via flow cytometer which produces results as geometric mean fluorescence intensity (MFI). The degree of phosphorylation of VASP will be directly proportional to the platelet inhibition by clopidogrel and is expressed as platelet reactivity index (PRI) derived from MFI [PRI(%) = 100 × (MFI_(PGE1)_ − MFI_(PGE1 + ADP)_)/MFI_(PGE1)_]. The main advantage associated with this assay is that it is highly specific for the P2Y12 receptors.^44^

### 4.3 Point of care tests

Point of care tests (POCTs) are tests that can be performed on whole blood at or near the bedside of the patient, more easily, such that rapid results can be obtained without pipetting and pretreatments to the sample. Frequently, VerifyNow P2Y12 assay, Multiplate assay, PFA‐100, Plateletworks, TEG, and ROTEM are designed as POCTs and used to monitor the effect of clopidogrel. POCTs are developed to overcome the limitations associated with LTA, such as labor intensiveness, cost, and time. POCTs are generally at high cost than normal LTA, but they have the ability to provide clot quality and monitors clot formation and progression even after the point of clot formation.^35^, ^53^, ^54^

#### 4.3.1 Rotational thromboelastometry: ROTEM delta and ROTEM platelet

Blood collected within 4 hours into 3.2% sodium citrate is needed to be incubated at 37°C before examination. ROTEM delta uses viscoelastic measurement. Blood is filled into the cuvette and a cylindrical pin, known as the sensor, is immersed into the cuvette so that there is 1‐mm gap between pin and cuvette wall, bridge by the blood. Sensor is rotated sideways by a spring. The pin rotates till the blood is in liquid form; however, when blood starts clotting, rotation of the pin slows down. Kinetic motion of the pin is detected mechanically and computation is expressed via thromboelastometric curve and numerical parameters. ROTEM delta has the ability to identify hypo‐ and hyperfunctional stages of clotting process. ROTEM can measure the parameters: clotting time, clot formation time (CFT), maximum clot firmness (MCF), and alpha angle, which use the angle between middle axis and tangent to the clotting curve through 2‐mm amplitude point. ROTEM has special techniques to monitor the coagulation pathway via intrinsic and extrinsic pathways, heparin action, and clot firmness after blocking hyperfibrinolysis by aprotinin. There are many variables that can be measured by ROTEM analysis. Commonly used parameters are coagulation time, CFT, α‐angle, amplitude at 10 minutes after CT, MCF, maximum lysis (ML), EXTEM (measures extrinsic pathway), APTEM (measure clot firmness after blocking hyperfibrinolysis), FIBTEM (measure clot firmness after blocking the platelets), INTEM (measures intrinsic pathway), and HEPTEM (same as INTEM, but inhibits heparin).^55^, ^56^, ^57^, ^58^, ^59^ Assay should solely depend on the instructions provided by the manufacturer as the results may vary depending on the temperature, reagent status, sample stability, and instrument status.^57^ In monitoring the effect of clopidogrel, ROTEM analysis could be performed using EXTEM and FIBTEM reagents. Thus, produces the results CT, MCF, and CFT of EXTEM and FIBTEM, respectively.^56^

However, ROTEM delta lacks the ability to monitor antiplatelet drug therapy due to increased production of thrombin during the assay. Thrombin thus produced stimulates platelets, preventing the detection of platelet inhibition by antiplatelet drugs. In order to overcome the above limitation, ROTEM platelet was combined to ROTEM delta such that single sample can be analyzed simultaneously with two techniques. ROTEM platelet consists of two channels which allow whole blood analysis via impedance principle. Sample cuvette is inserted into temperature‐controlled channel and then two wires of the electrodes are placed inside the cuvette. When the activated platelets aggregate around the surface of the electrode wires, impedance between wires increases producing an impedance curve. The magnetic stirrer inside the cuvette prevents the deposition of blood cells at the incubation. If the patient is effectively responding to clopidogrel therapy, the curve lies below the reference curve. Impedance curve provides three parameters, where area under curve (Ω × min) gives overall platelet aggregation, amplitude at 6 minutes (Ω/min) gives degree of platelet aggregation after activation, and maximum slope (Ω) indicates time taken for platelet aggregation. ROTEM platelet was specifically designed to monitor antiplatelet therapy by three assays. ARATEM assay monitors aspirin therapy by activator arachidonic acid, ADPTEM assay monitors clopidogrel using ADP, and TRAPTEM monitors thrombin by thrombin‐receptor activating peptide‐6.^56^, ^60^

#### 4.3.2 Thromboelastography

Thromboelastography is a rapid POCT performed on citrated whole blood. This technique quantitatively measures the viscoelastic properties of the platelets in forming the platelet plug. The principle will be same as that of ROTEM platelet, which has a disposable cup with a detection pin fixed at the center. Difference is that the TEG rotates the cup and ROTEM rotates the pin first when the clot forms. Prior to clotting, blood has the minimum viscosity. Therefore, the waves of the cup cannot induce the movement of the pin. When blood starts to coagulate, blood viscosity increases, and the clot gets attached to both the cup and pin causing the movement of the pin upon induced by the cup. When the viscosity increases gradually, amplitude of the pin movement also increases. Once the fibrinolysis starts, platelet clot dissolves decreasing the blood viscosity. Change of amplitude is expressed graphically against time.^61^

Conventional TEG lacks the ability to determine the ADP receptor inhibition for the reason that the excess production of thrombin through the technique. Conventional TEG will show normal maximum amplitude for a patient under clopidogrel therapy who was shown to have an ultimate platelet inhibition via LTA, which was considered as a major problem associated with conventional TEG. ROTEM expresses CT, α‐angle, CFT, MCF, and clot lysis, whereas those parameters are expressed as reaction value (*R* value), α‐angle, *K*‐value, maximum amplitude, and amplitude at 30 minutes (LY30) in TEG. However, by overcoming the above problem, modified TEG has the capability to monitor the clopidogrel action, without thrombin generation. ADP is used as the agonist to measure the degree of platelet aggregation of patients under clopidogrel therapy.^58^

#### 4.3.3 VerifyNow P2Y12 assay

VerifyNow assay is performed as a POCT on citrated whole blood as a turbidometry assay. When PGE1 is introduced, ADP induces platelet coagulation and aggregates with the help of fibrinogen‐coated beads. Most important fact is that, the assay is sensitive and specifically targets the P2Y12 receptors while inhibiting the action of P2Y1. As in LTA, the platelet aggregation is determined by the percentage of the light transmission and expressed in PRU (P2Y12 reaction units). Low PRU indicates the high P2Y12 receptor inhibition and better response to clopidogrel. Measurement of inhibition of P2Y12 receptor inhibition as a result of thrombin receptor‐activating peptide‐induced platelet aggregation has become an added advantage. Percentage inhibition is reported as [Base PRU − Posttreatment PRU]/[Baseline PRU] × 100. VerifyNow assay is a rapid test which can be performed even at bedside within 5 minutes, which has been an advantage when compared with LTA and VASP phosphorylation assays. VerifyNow assay also has the capability to monitor the effect of clopidogrel on P2Y12 receptors directly thereby helps to determine the adequacy of the loading dose of clopidogrel in patients who will be subjected to coronary stenting. Further, the assay has a simple technique and interpretation of results can be done easily.^51^, ^62^, ^63^ Cut‐off value for the suboptimal clopidogrel response in terms of percentage aggregation is ≥70% for 10µM ADP and ≥50% for 5µM ADP.^49^

#### 4.3.4 Platelet function assay‐100

Platelet function assay‐100 is another point of care assay to monitor the action of clopidogrel. This can be performed on less volume of citrated whole blood. This assay monitors the platelet aggregation and effect of antiplatelet drugs under higher shear stress. Hence, this assay can be performed rapidly in less time using less labor which is an added advantage when compared with conventional aggregation assays. PFA‐100 has cartridges coated with collagen and epinephrine or ADP. Blood drawn from the patient who is under clopidogrel therapy will flow under higher shear rate through the capillary and a small aperture of PFA‐100 analyzer toward the coated cartridge. Platelets will aggregate and form the ADP‐induced platelet plug by blocking the aperture. The time taken for complete occlusion of the aperture is recorded as closure time (CT). Collagen‐ADP (CADP) cartridge measures the platelet dysfunction due to clopidogrel. Prolonged CT indicates the better response to clopidogrel.^64^, ^65^, ^66^, ^67^ CT for CADP cartridge is 55‐137 seconds.^68^

#### 4.3.5 Multiplate analyzer

Multiplate analyzer is a sensitive, novel, point of care platelet function analyzer which can be performed on citrated whole blood using electrical impedance aggregometry principle. The main aim of the assay was to monitor the platelet function inhibitors. When the samples obtained from the patients who are under clopidogrel therapy are exposed to ADP agonist, platelets will get stimulated and results in ADP‐induced platelet aggregation. Once the aggregated platelets attach tightly to the sensor wires in the Multiplate device, an electrical resistance will be created between the wires. ADP with 200‐μmol/L concentration interacts with P2Y1 and P2Y12 receptors inducing irreversible aggregation. However, ADP + PGE1: 200 μmol/L + 300 nmol/L inhibits aggregation by P2Y1 receptor, thus increasing specificity for P2Y12 receptors. Change in the impedance can be shown by the graph; arbitrary aggregation units (AU) against time. In addition, the three parameters, area under the curve, height of the curve, and maximum slope, express the complete platelet reactivity, platelet aggregation, and velocity, respectively.^69^, ^70^

#### 4.3.6 Plateletworks analyzer

Plateletworks is a POCT device performed on whole blood. Plateletworks analyzer measures the platelet count of the sample before the addition of the ADP agonist and then after platelet aggregation upon addition of ADP. Usually, reference platelet count taken from the K3‐EDTA anticoagulant blood without ADP and other count is taken from citrated anticoagulant sample with ADP agonist.^71^

In normal patients, the platelets get aggregated in the presence of agonist and the resultant platelet count is considered to be zero. When the blood flows through the aperture, the constant electric current will develop an electrical pulse which is amplified and converted to obtain the platelet count.^72^ When the platelet aggregates exceed the threshold limit for platelet size, such platelets are not considered for the resultant platelet count. Percentage inhibition of platelet aggregation provides the degree of platelet aggregation in the presence of clopidogrel. Percentage of inhibition is expressed as the ratio between platelet counts before and after exposure to ADP. Plateletworks is widely used for monitoring the effectiveness of antiplatelet drugs such as clopidogrel and aspirin.^53^, ^73^, ^74^, ^75^ Blood samples should be analyzed within 10 minutes to obtain accurate results. There are studies to prove the significant correlation between Plateletworks and LTA. However LTA determines only macroaggregation while Plateletworks is sensitive for microaggregation. Minimum studies have been conducted to show the prediction of outcomes of cardiovascular diseases.^71^, ^73^, ^76^ In the determination of the cut‐off values for Plateletworks assay, it had shown 63% sensitivity and 58.5% specificity using ROC curve analysis.^74^

### 4.4 High‐performance liquid chromatography

High‐performance liquid chromatography is generally used for identification and quantification of chemical compounds. HPLC consists of a stationary phase which is packed either with a resin or silica gel beads and mobile phase. Methanol and acetonitrile are used as solvents for the separation of analyte. Molecular interaction between stationary phase, analyte, and solvent decides the retention time. Separation of analytes in normal HPLC depends on the polarity where stationary phase is polar and mobile phase is nonpolar. However, in reverse phase HPLC, the separation is based on hydrophobic interactions between polar mobile phase and nonpolar stationary phase. During the separation and quantification of active metabolites, the internal diameter of the HPLC column, particle size, and pore size of the stationary phase and pump pressure should be considered for optimum performance.^77^

Around 5%‐40% of patients who are under clopidogrel therapy may show resistance to clopidogrel and the cause may be due to impaired drug absorption by the hepatic cells or impaired drug metabolic activity. However, the cause can be clearly explained via analysis of drug metabolite levels in the patient's blood via HPLC method. However, due to low levels of the prodrug in plasma after ingestion and instability of thiol derivatives, it has become difficult to use HPLC method for determination of drug levels in patient plasma.^78^ Hence, most of the studies were conducted using plasma of healthy volunteers and spiking the samples with prepared concentrations of clopidogrel.^79^, ^80^ Thereby, those studies were only able to determine either the prodrug only or one of the derivates from CTM or CLPM. One such study was able to analyze the pharmacokinetic properties of only CTM isomers, but not the isomers or prodrug or CLPM. They have analyzed the H1‐H4 isomers using a reverse‐phase ultra‐high‐performance liquid chromatography and tandem mass spectrometry (MS) with a concentration accuracy for a range of 0.5‐250 ng/mL.^81^

Once the HPLC technique is validated by the means of the parameters linearity, stability, precision, and accuracy, it was identified as the most sensitive, specific analysis method to monitor the effect of clopidogrel on patient responsiveness.^82^, ^83^ Two studies have been found to analyze the clopidogrel and its metabolites in patients with myocardial infarction and PCI. One study has determined the plasma levels of clopidogrel, inactive carboxyl metabolite, and active H4 thiol metabolite in the plasma of ST‐elevated myocardial infarction patients.^11^ The second study studied the plasma levels of clopidogrel, CLPM, active H4, and inactive H3 of patients under PCI. The study has found that the maximum absorption of 75 mg of drug was 2 ng/mL in 1.4 hours and 300 mg of drug was 4.5 ng/mL in 1.2 hours by intestines. Platelet aggregation with 75 mg dose was found to be between 37 and 747 AU/min. The significant correlation between maximum concentration of H4 isomer and platelet aggregation depicts the importance of use of both the above parameters in the detection of response to clopidogrel therapy.^78^

### 4.5 Pharmacogenetic assays

CYPA1, CYP2B6, and CYP2C19 are CYP_450_ enzymes that participate in the first step of converting clopidogrel prodrug to 2‐oxo‐clopidogrel. The second step which forms the thiol metabolite is catalyzed by the enzymes CYP2B6, CYP2C9, CYP3A4/5, and CYP2C19. CYP2C19 is considered as the main enzyme involved in the whole process as it provides 44.9% to the first step and 20.6% to the second step. In addition, CYP3A4 too contributes 39.8% to the second step.^84^, ^85^ Hence, bioactivation process of clopidogrel could be altered due to genetic polymorphisms of the genes *CYP2C19*, *CYP3A4/5*, *CYP2C9*, and *CYP2B6* that code for their respective enzymes. Out of these genes, *CYP2C19* gene polymorphism is studied mainly due its association with both the steps. *CYP2C19* is highly polymorphic, where *1/*1 allele is considered as normal and two loss of functional (LoF) alleles due to single nucleotide polymorphism are (*2) and (*3). These LoF alleles result in poor clopidogrel therapeutic outcome. Although *CYP2C19* and *CYP2C9* LoF cause poor clopidogrel outcome, *CYP1A2*,*CYP2B6*, and *CYP3A4/5* alleles have not shown any considerable effect on clopidogrel effect. The Clinical Pharmacogenetic Consortium has developed a guide to interpret CYP2C19 genetic test. It explained that *1/*17, *1/*17, and *17/*17 genotypes show normal enzyme activity and clopidogrel 75 mg daily dose could be administered. Genotypes *1/*2, *1/*3, and *2/*17 indicate intermediate enzyme activity and genotypes *2/*2, *2/*3, and *3/*3 show absence of enzyme activity. Patients with these genotypes should be treated with either Prasugrel 10 mg daily or Ticagrelor 90 mg twice a day.^86^ Prasugrel and Ticagrelor are used for clopidogrel poor responders as these are not affected by *CYP2C19* LoF allele.^87^ It was found that CYP2C19*2 resulted in poor platelet inhibition by clopidogrel and hence causes poor outcomes in PCI patients.^88^ CYP2C19*17 gene polymorphism too can be studied which results in enhanced enzyme activity due to increased gene transcription.^89^ Clopidogrel absorption in the intestine is controlled by a transporter protein called P‐glycoprotein multidrug resistance‐1, which is coded by the gene *ABCB1*. Polymorphism of *ABCB1* gene has shown poor absorption of clopidogrel by intestines.^90^ *CES1* is another genetic polymorphism which could be studied as the CES1 enzyme synthesized by the gene results in the metabolism of clopidogrel. LoF of this allele causes increased concentration of active thiol metabolite,^91^ whereas polymorphism of *PON1* gene reduces the enzyme activity of PON1 esterase and results in reduction of active thiol metabolite concentration.^92^ Gain of functional haplotypes *H2* of *P2Y12* gene increases atherothrombotic events and affects the action of clopidogrel.^93^ However, due to the difficulty in predicting the outcome of every genetic variation associated with clopidogrel, Clinical Pharmacogenetics Implementation Consortium guidelines for *CYP2C19* genotype and clopidogrel therapy have not endorsed to follow *CYP2C19*‐guided therapy.

Several pharmacogenetic tests are available to determine clopidogrel response by analyzing different *CYP2C19* variants. Whole blood is used for polymerase chain reaction and primer extension reaction, which is the underlying technique of the tests AccuType^™^ CP and Cytochrome P450 2C19 10 mutations assay (ARUP Laboratories, Salt Lake City, UT, USA). This technique is used by clopidogrel efficacy test (by 23andMe Company, Sunnyvale, CA, USA); however, it is performed for salivary samples. Medication panel (Navigenics, Inc, Foster City, CA, USA) and drug response test (Pathway Genomics, San Diego, CA, USA) use salivary samples on two different techniques: TaqMan SNP genotype assay and DNA testing, respectively. DNA testing is an underlying principle in many other tests such as Clopidogrel genetic test (TheranostiCs Lab, Auckland, New Zealand), CYP450 2C19 (Plavix^™^) gene test for buccal swabs and Verigene CYP2C19 XP/CYP2C19 nucleic acid test. PlaVitest by Genelec Corporation can be performed on either whole blood or buccal swabs using extended CYP2C19 DNA mutation panel.^94^ CYP2C19*2 genetic test produced by Spartan Biosciences is a rapid test which could be performed within 1 hour on buccal swab.^87^ Clopidogrel antiplatelet effect was studied by using a rapid test called Rapid Infinity analyzer. DNA extracted from the whole blood collected in EDTA was hybridized to BioFilmChip microarray in the analyzer, hence can be used to determine CYP19*2, *4 and CYP2C9*2, *3 polymorphisms.^95^ Rapid Verigene and classical TaqMan assays have been compared by analyzing CYP2C19*2, *3, *4, *5 and *17 polymorphisms. Study have found that turnaround time, labor requirement, and relative cost is less in the Verigene assay than TaqMan assay, although reagent cost of Verigene assay is considerably high. Hence, Verigene is considered having better performance.^96^

## 5 COMPARISON OF THE PFTS

Light transmission aggregometry is the most acceptable method to determine the validity of other novel PFTs. In contrast to these advantages, the major disadvantage is that the higher concentrations of ADP agonist have the ability to induce the platelet aggregation without altering the shape of platelets. Other drawbacks of this assay are listed in Table 2.^25^, ^44^, ^97^

**FIGURE 3:**
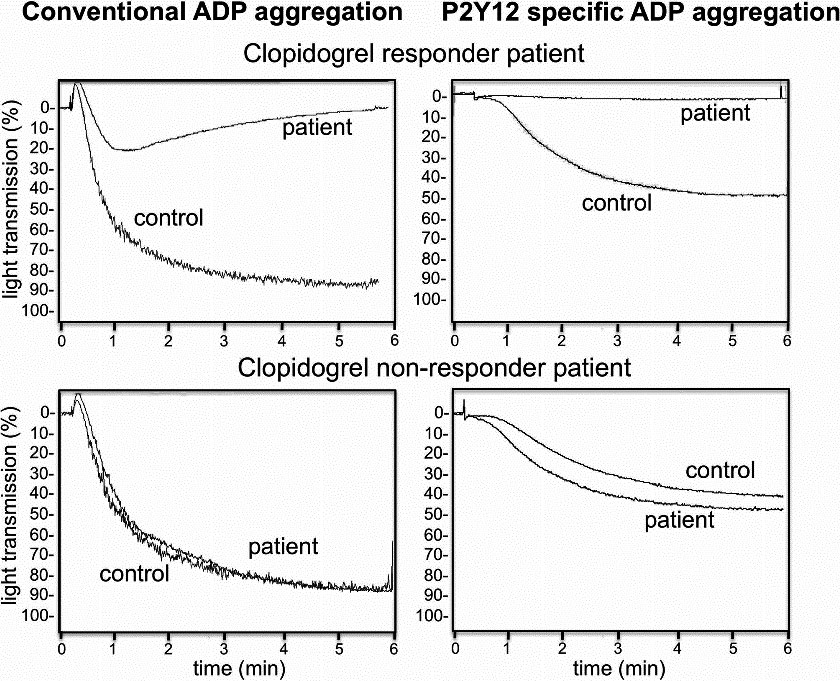
Differentiation of results obtained from conventional and modifies light transmission aggregometry performed on clopidogrel responder and non‐responder patients (Bagoly et al, 2013).^44^Conventional adenosine diphosphate (ADP) aggregation (without prostaglandin E1 [PGE1]) is shown in the left side and P2Y12 specific ADP aggregation with PGE1 is shown in right side. Top graphs shows the samples obtained from clopidogrel responder patients and below shows the samples obtained from clopidogrel resistant patient

A study conducted has shown that the traditional method of LTA cannot be used as a test to monitor the effect of clopidogrel. Conventional ADP represents platelet aggregation due to both P2Y1 and P2Y12 receptors, whereas modified method with PGE1 shows the platelet aggregation specific to PGE1 (Figure 3). Further, when comparing the VerifyNow P2Y12 assay, Modified ADP/PGE1 assay, and VASP phosphorylation assay, there was a 12%‐54% variation in the cut‐off values for clopidogrel nonresponsiveness between the above assays. Percentage of clopidogrel nonresponders detected by modified AD/PGE1 aggregation method and VerifyNow P2Y12 assay was much similar. When monitoring the clopidogrel active metabolite of clopidogrel in the plasma of patients, VASP assay was identified as more sensitive as the assay is more specific for P2Y12 assay. This study has concluded that the highest correlation was shown between the ADP/PGE1 aggregation assay and VASP phosphorylation assay and less correlation between conventional LTA and VerifyNow assay and VASP phosphorylation assay in monitoring the efficiency of action of clopidogrel.^44^ POCTs, such as VerifyNow P2Y12 assay, PFA‐100, Impact cone and Platelet analyzer, and Thromboelastograph Platelet Mapping System, were developed to overcome the limitations of the LTA technique although it is considered as the gold standard test.

**FIGURE 4:**
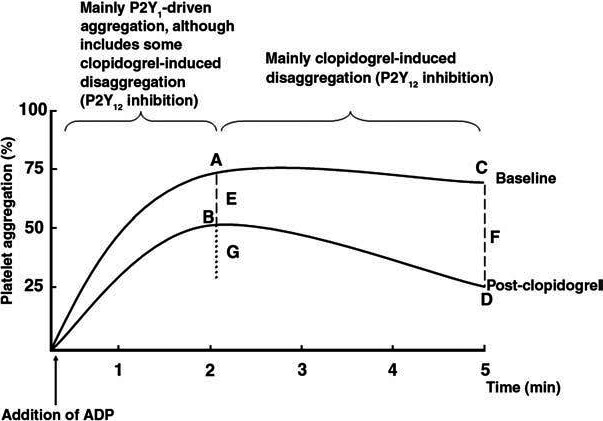
ADP‐induced platelet aggregation curve obtained from light transmission aggregometry before and after clopidogrel therapy (as cited by Lordkipanidze et al, 2009).^99^ADP, adenosine diphosphate

A study conducted to compare the effectiveness of LTA, modified TEG, and PFA‐100 has recruited 28 patients on clopidogrel therapy. The correlation between the LTA and TEG results was considerably acceptable (*κ* = 0.81). PFA‐100 had shown variable results for only two individuals who have been detected as resistant to clopidogrel by LTA and hence no relation was shown with either TEG or LTA. However, PFA‐100 was not recommended to demonstrate considerable difference in clopidogrel responses, but may be useful to demonstrate aspirin responses.^64^ As defined by the upper curve, Figure 4, ^46^ depicts the platelet aggregation as a result of activation of P2Y1 receptor (till Point A). After point A, the platelet aggregation becomes stabilized due to the activation of P2Y12 receptors and the end point is noted as point C. Hence, points A and C are more likely in the same level due to the absence of clopidogrel. When clopidogrel is administered, the stabilization of P2Y12 decreases, initiating the disaggregation of the platelets, indicated by end point D. Absolute change in the peak level of the curve (Line E—absolute inhibition at peak aggregations) and change in the levels of end points (Line F—absolute inhibition at late aggregations) define the degree of nonresponsiveness to clopidogrel. Line G provides a measure of disaggregation. Points A and B indicate the peak points of platelet aggregation before and after clopidogrel administration, respectively. Points C and D reflect the late aggregation before and after clopidogrel administration, respectively.^46^, ^98^

Both TEG and ROTEM have the capability to analyze the physical properties of the clot including the clot stability and strength, fibrin formation, clot formation, and clot lysis, but not thrombolysis or fibrinolysis. Moreover, both platelet function and defects associated with coagulation cascade are also monitored. Controversial result was obtained from the studies,^100^, ^101^, ^102^ that TEG has a very low ability in predicting the hemorrhage and guiding the transfusion of blood products, whereas. However, TEG platelet mapping can predict the excess hemorrhage in patients undergoing coronary artery bypass and under clopidogrel and aspirin therapy.^103^ The patients who were under clopidogrel therapy for 5 days prior to bypass were shown 70% cut‐off value for platelet responsiveness for clopidogrel and had the capacity to determine the hemorrhage level at the surgery and transfusion guide.^104^, ^105^

Vasodilator‐stimulated phosphoprotein phosphorylation assay has a higher ability to determine thienopyridine‐induced inhibition of platelets when compared with turbidimetric platelet aggregation assays. Flow cytometric analysis was able to define the associated clinical situation in detail. However, epinephrine action on α2A receptors can initiate the dephosphorylation of VASP, and nitric oxide donors can induce the VSP phosphorylation through cGMP causing false results in the analysis of the clopidogrel action.^106^, ^107^, ^108^ GRAVITAS trial (Gauging Responsiveness with a VerifyNow P2Y12 Assay: Impact on Thrombosis and Safety) has setup the cut‐off value for clopidogrel high on‐treatment platelet reactivity (HTPR) as ≥208 P2Y12 PRU, which has been found in more than half of the selected subjects and has shown a >5‐fold increased risk of cardiovascular death, myocardial infarction, and stent thrombosis at 60 days.^109^

Significant correlations were found between multiplate analyzer, PFA‐100, and LTA after ADP‐induced platelet aggregation in samples with clopidogrel therapy. However, sensitivity (78%), specificity (95%), accuracy (92%), positive predictive value (80%), and negative predictive value (95%) of Multiplate assay are significantly higher (<0.0001) than LTA.^110^ Multiplate assay keeps the cellular environment unchanged, hence the assay becomes more rapid.^111^ Many studies have observed that PFA‐100 is more sensitive in determining the effect of clopidogrel rather than aspirin.^112^ However, a study has shown that PFA‐100 cannot be used in the determination of residual antiplatelet activity of clopidogrel. The actual platelet resistance in clopidogrel‐related HTPR can be detected by PFA‐100 when compared to aspirin.^113^

Light transmission aggregometry had shown 60% sensitivity and specificity in HTPR once compared with VASP assay. Although both the assays have a high negative predictive value (94%), they lack a proper standardization technique.^114^ The cut‐off value for clopidogrel responsiveness when measured with Multiplate analyzer was found to be >416 AU/min under 84% and 70% sensitivity and specificity, respectively.^115^

Although there are many platelet function assays, none of the tests have been optimized or fully standardized to study the overall effect of clopidogrel and patient responsiveness to the drug. Reason is that, PFTs have their own pros and cons (Table 1).^36^, ^122^

**TABLE 1:** Advantages and disadvantages of PFTs used for monitoring of clopidogrel effect

PFT	Advantages	Disadvantages
**VASP phosphorylation assay** Principle: Flow cytometry	Provides detail explanation on platelet glycoprotein receptors Specific to assess the P2Y12 receptor inhibition Perform on whole blood Stable results can be obtained even after 24 h of sample collection at room temperature Low sample volume Can monitor peak plasma levels of active thiol metabolite of clopidogrel. So, real in vivo biological activity of clopidogrel can be measured/more physiological Possible to separate the “Normal” group from the patients with platelet inhibition effect Not affected by platelet count. Hence, suitable for thrombocytopenic patients	More time‐consuming Need special expertise to perform Cannot perform at/near bedside Cannot produce rapid results Expensive as it needs a flow cytometer Special pretreatment to sample is required along with pipetting Difficult to perform routinely Cannot measure glycoprotein IIb/IIIa receptor Affected by artifacts
**LTA** Principle: Turbidimetric‐based optical detection of platelet aggregation under low shear rate	Can be used to validate other novel platelet function tests Possible to adjust the instrument to obtain many parameters Good predictivity of clinical situation Many studies are available to prove the efficacy of the assay, hence considered as gold standard Measure overall platelet function and platelet surface glycoprotein including acquired and inherited defects Monitoring clopidogrel drug effect	Complex and time‐consuming Poor standardization of the technique Perform only platelet‐rich plasma. So, sample preparation steps are available; centrifugation and pipetting steps Need more sample quantity Cannot be performed at/near bedside Operator errors affect the results Measures AMC of clopidogrel only under high concentration of ADP (20 µmol/L) Significant correlation with peak levels of AMC cannot be found with low ADP concentration (5 µmol/L). So, less sensitive to define clopidogrel responsiveness Highly affected by pre‐analytical variables such as diet, hematocrit, operator errors, age, and gender Results are affected by platelet count and not suitable for thrombocytopenic patients P2Y1 receptors associated platelet aggregation will be induced under low‐dose ADP, unless they are blocked by PGE1 Assay performed under low shear rate. Hence, nonphysiological
**VerifyNow P2Y12 assay** Principle: Turbidimetric assay	Fully automated point of care device Can be performed with whole blood Produce rapid results Simple technique. No expertise labor is required. No pipetting, centrifugation steps, and sample processing Small sample volume is required. Widely used for monitoring clopidogrel effect Results correlate considerably with LTA and other platelet function‐POCTs Many disadvantages associated with LTA are addressed and rectified in the assay Able to monitor clopidogrel efficacy and plasma levels of active thiol metabolite Commonly used for monitoring the dual therapy with aspirin and clopidogrel Assay is more physiological Can be used for routine analysis Most suitable device identified thus far to use as a POCT	Assay cannot be adjusted to obtain different parameters or to predict the clinical condition more accurately Cannot assess the other physiological platelet activation pathways High cost for cartridges Does not provide percentage inhibition of receptor or activity Occasional failures in channels may occur Though results can be delivered within 5 min, it is recommended to incubate the sample for 10 min for optimum results Affected by hematocrit, platelet count, triglyceride, and fibrinogen levels
**PFA‐100** Principle: Platelet aggregation is measured as the time required for closure of the aperture in the cartridge under high shear stress	High sensitivity Simple technique Whole blood is used 3.8% sodium citrate anticoagulant will provide higher stability for CT results Fully‐automated, point of care device No sample pretreatment steps Cartridge (CADP) is sensitive to measure P2Y12 receptor Small volume of sample is needed Produce rapid results Standardized technique Able to screen defects associated with primary hemostasis More physiological than LTA High negative predictive value Insensitive to clotting factor deficiencies More sensitive PFA‐P2Y cartridge has been developed which is more specific than collagen/ADP cartridge Able to diagnose inherited and acquired platelet defects, bleeding, and thrombotic risk Used to monitor antiplatelet therapy, mainly the effect of clopidogrel drug	Assay cannot be adjusted Requires sample pipetting only Results may highly vary depending on the hematocrit level (<50 × 109/L and 25%) and vWF levels Assay is affected by citrate concentration hematocrit, platelet count, certain drugs, certain food, and acquired platelet function defects Less studies have conducted to prove the effectiveness of the assay Collagen/ADP cartridge is relatively insensitive to thienopyridine effect. Hence, does not correlate with clopidogrel therapy PFA‐P2Y cartridge is only available for research purposes Not recommended to measure glycoprotein IIb/IIIa receptor
**Multiplate analyzer** Principle: Impedance aggregometry	Whole blood Simple technique Rapid results, within 10 min Standardized procedure Point of care device Less pretreatments to the sample More physiological Provides better correlation with LTA Sensitive to monitor the clopidogrel effect Able to monitor platelet surface glycoprotein receptors	Semi‐automated Samples should be analyzed as soon as collection. Novel method. So, not much clinical studies to prove the effectiveness and evaluating the predictive value of the results obtained. Need more sample volume Expensive Depend on hematocrit and platelet count
**TEG/ROTEM delta and ROTEM platelet** Principle: Measures viscoelasticity under low shear stress	Small volume of whole blood Produce quick results within 5‐10 min. ROTEM platelet provides results within 6 min Point of care device Provide details regarding fibrinogen and clotting factors Able to differentiate platelet disorders and clotting abnormalities Measures the rate of clot formation and stability Used to assess hemostasis worldwide ROTEM platelet can monitor the effect of clopidogrel and other antiplatelet drugs	Require labor expertise and expensive Does not monitor warfarin effect Artifacts in fibrin channel may affect percentage ADP estimates Need calculations with three imprecise variables (CV ~ 20%). Therefore, low precision. Co‐efficient of variance for TEG is 7.1%‐39.9% and ROTEM is 7%‐83.6% according to UK‐NEQAS data TEG and ROTEM delta are not ideal test to monitor the effect of clopidogrel and other drugs Requires pipetting Results may be affected by the operator errors^123^
**Plateletworks** Principle: Impedance aggregometry—Measures platelet count before and after addition of ADP	POCT Small volume of citrated whole blood Monitoring the response of platelets to antiplatelet therapy Easy to perform No sample preparation No interaction of platelets with red cells and white cells Can monitor clopidogrel effect and dual therapy along with aspirin Glycoprotein IIb/IIIa receptors of platelets and predict outcomes	Limited studies for assessing the effectiveness of the assay More time due to sample preparation Not standardized to define the antiplatelet resistance Should perform within 10 min Affected by pre‐analytical variables Does not have the ability to measure aggregation directly Require adjunctive platelet count
**Pharmacogenetic assays**	Predict the therapeutic outcome of clopidogrel as well as other drugs such as omeprazole, diazepam, and anti‐seizure drugs Aid in genotype‐guided therapy where necessary Determine the possible drug reactions *CYP2C19* polymorphism specifically determines clopidogrel efficacy Whole blood, buccal swabs, and saliva can be used based on the test used No patient preparation	Expensive than PFTs More time required for most techniques except for rapid genetic tests Complex techniques which require skilled labor Lack of studies to prove the relative cost‐effectiveness and efficacy to determine clopidogrel effect completely Difficult to use routinely for every patient. Usually performed in high‐risk patients for poor response to standard dose of clopidogrel usually after PCI^94^ or if there is excess bleeding even after medication Limited studies to prove the correlation between the results of different types of genetic tests available and with other PFTs *CYP2C19*‐guided therapy is not recommended to practice by ACA, AHA, and SCAI as clopidogrel nonresponsiveness may be associated with other genetic and nongenetic factors^85^

Abbreviations: ACA, American College of Cardiology; ADP, adenosine diphosphate; AHA, American Heart Association; AMC, active thiol metabolite of clopidogrel; CV, coefficient of variation; LTA, light transmission aggregometry; PFA, platelet function assay; PGE1, prostaglandin E1; POCT; point of care test; ROTEM, rotational thromboelastometry; SCAI, society for cardiovascular angiography and interventions; TEG; thromboelastography; UK‐NEQAS, United Kingdom National External Quality Assessment Service; VASP, vasodilator‐stimulated phosphoprotein; vWF, von Willebrand factor.

By comparing the results obtained from the study, it was concluded that VASP phosphorylation assay, VerifyNow P2Y12 assay, and LTA with 20 µmol/L ADP are ideal PFTs to monitor clopidogrel responsiveness.^54^, ^66^, ^70^, ^117^, ^124^ When compared with LTA, VASP‐P assay was more suitable^125^ and out of LTA, Multiplate assay, Verify Now assay, and TEG, Multiplate assay was found to be ideal to monitor the clopidogrel.^126^ Limitations of LTA has paved the way for the development of standardized PFTs such as PFA‐100, Multiplate, and VerifyNow P2Y12 assays.^36^

### 5.1 Reference ranges

High on‐treatment platelet reactivity on clopidogrel was defined by ROC curve analysis for VASP phosphorylation assay (PRI > 50%), VerifyNow P2Y12 assay (>235‐240 PRU), LTA (>46% for 5 µmol/L ADP), and Multiplate analysis (>468 arbitrary AU/min).^67^, ^127^, ^128^ Table 2 indicates the reference ranges to determine the clopidogrel responsiveness by different PFTs.

**TABLE 2:** Reference ranges for clopidogrel responsiveness

Assay	Reference range
PFA‐100	55‐137 s for CADP cartridge
VerifyNow P2Y12 assay	>180‐376 PRU indicates that drug is not available in blood 10‐180 PRU indicates decreased platelet reactivity to P2Y12 inhibitor
ROTEM delta/TEG/ROTEM platelet	Consider the impedance curve provided by ROTEM. Reference ranges for CT (s), CFT (s), α‐angle (°), A10 (MM), A20 (mm), MCF (mm), LI 30 (%), and ML (%) within 1 h For EXTEM: 38‐79 s, 34‐159 s, 63°‐83°, 43‐65 mm, 50‐71 mm, 50‐72 mm, 94%‐100%,, and <15%, respectively. For FIBTEM: A10 is 7‐23 mm and A20 is 8‐24 mm^56^, ^129^
LTA	Percentage optical density vs time graph will provide platelet aggregation traces. Based on the pattern, disease condition could be interpreted. Commonly encountered cases are Glanzmann's thrombasthenia, Bernard‐Soulier syndrome, storage pool disorder, the effect of aspirin, and the effect of clopidogrel
Multiplate analyzer	Interpretation is same as LTA
VASP Phosphorylation assay	Need to study the scatterplot graphs to interpret the condition
Plateletworks	Provides percentage aggregation

Abbreviations: A10, amplitude at 10 s; CFT, clot formation time; clotting time; CT; LI, lysis index at 30 min; MCF, maximum clot firmness; ML, maximum lysis; PFA; platelet function assay; PRU, platelet reactivity units; ROTEM, rotational thromboelastometry; TEG; thromboelastography.

## 6 SUGGESTED IMPROVEMENTS FOR PLATELET FUNCTION TESTING

An anticoagulant, such as hirudin, should be used instead of the 3.2% trisodium citrate when possible. The anticoagulant used must not interfere with the normal physiology of the sample.^54^, ^130^ An advanced principle with a simple technique which can be used globally for different agonists without altering the physiological environment of the sample would be ideal.^67^ Further, PFT should have the capacity to determine not only platelet aggregation, but also to measure the procoagulant activity of platelets. Most importantly, reference ranges need to be established to differentiate the normal from different clinical conditions and also identify low‐/high‐risk patients for clopidogrel and other antiplatelet drug nonresponsiveness. When the current clinical requirements are considered, the need for the PFT to be used as a screening test has increased, in order to obtain results and quick decisions.^131^ It would be better to develop more standardized and quality‐controlled PFT, minimizing the effect from pre‐analytical errors to the final result of the assay in addition to being cost‐effective. Assays more specifically assess the metabolism of clopidogrel and its thiol metabolites will help to study the increasing patterns of patient nonresponsiveness to clopidogrel. Performing large randomized clinical trials in different populations will benefit to identify the clinical outcomes of monitoring the effect of clopidogrel on individuals. At present, POCT assays, which use non‐anticoagulated finger prick blood with disposable cartridges, are available (such as PlaC or PFT).^66^ POCT‐PFTs should be developed to monitor the clopidogrel action on platelet inhibition via biomarkers, such as CD^34+^, which can be found in endothelial progenitor cells, chemokine CXCL12 (stromal cell‐derived factor‐1α, SDF‐1α), produced by platelet and stored in α granules. In future, there is a possibility for the development of sequencing assays to be used widely as screening tests to determine coagulation and hemorrhagic disorders.^132^

## 7 CONCLUSION

In conclusion, at present no PFT has proved their optimum ideality for monitoring the platelet inhibition and efficacy by clopidogrel. Although new POCTs may be considered useful for the existing clinical setup, further prospective studies are required to determine the efficacy of them. For a PFT to become ideal, they should be able to use non‐anticoagulated whole blood to assess thrombus formation as wells as platelet aggregation/inhibition by clopidogrel. Further, assay should be able to perform on blood under high shear rate, to monitor in vivo thrombolysis and thrombus stability. Hence, the requirement for a new, ideal PFA as a point of care device which could overcome the limitations discussed has become an important need in the clinical setup globally. This would help in monitoring the effectiveness of clopidogrel on patients and helps to identify the responsiveness status and reasons for nonresponsiveness to the drug, thus aiding to develop proper treatment plans. Although determination of *CYP2C19* polymorphism is related to clopidogrel, it is not recommended to use *CYP2C19*‐guided therapy. Upon comparing the PFTs, VerifyNow P2Y12 assay seems to be the most suitable POCT for monitoring clopidogrel therapy at present. ROTEM platelet appears to have a good potential, however, lacks comparative studies. Performing PFT along with HPLC for clopidogrel levels in blood would provide a better understanding regarding clopidogrel therapy.

## DISCLOSURES

None declared.

## AUTHORS’ CONTRIBUTIONS

All the authors have made substantial contributions to conception and design, or acquisition of data, or analysis and interpretation of data. Involved in drafting the manuscript and revising it critically for important intellectual content. All authors gave the final approval of the version to be published. Each author have participated sufficiently in the work to take public responsibility for appropriate portions of the content, and agreed to be accountable for all aspects of the work in ensuring that questions related to the accuracy or integrity of any part of the work are appropriately investigated and resolved.

## Data Availability

Data sharing is not applicable to this article as no new data were created or analyzed in this study.
